# Identification and Verification of SLC27A1, PTBP1 and EIF5A With Significantly Altered Expression in Aggressive Pituitary Adenomas

**DOI:** 10.3389/fsurg.2022.923143

**Published:** 2022-06-21

**Authors:** Jianhua Cheng, Ruya Sun, Ding Nie, Bin Li, Song Bai Gui, Chu Zhong Li, Ya Zhuo Zhang, Peng Zhao

**Affiliations:** ^1^ Neurosurgical Department, Beijing Tiantan Hospital, Capital Medical University, Beijing, China; ^2^ Department of Cell and Biology, Beijing Neurosurgical Institute, Capital Medical University, Beijing, China; ^3^ Department of Biomedical Informatics, Department of Physiology and Pathophysiology, Center for Noncoding RNA Medicine, MOE Key Lab of Cardiovascular Sciences, School of Basic Medical Sciences, Peking University, Beijing, China

**Keywords:** aggressive pituitary adenom, TMZ, SLC27A1, PTBP1, recurrent pituitary adenoma, primary pituitary adenoma

## Abstract

**Background:**

Aggressive pituitary adenoma encircling the internal carotid artery has a poor clinical prognosis because of a high surgical risk and a high recurrence rate. This seriously affects patients’ quality of life and yet there is no effective medical treatment. The European Diagnostic Guidelines have recommended the use of temozolomide (TMZ) for these aggressive pituitary adenomas, but the treatment remission rate has been less than 50%.

**Methods:**

In this study, transcriptome sequencing of pituitary tumour tissues and TMZ-treated pituitary tumour cell lines were employed to explore the significance gene expressions affecting the efficacy of TMZ treatment for pituitary tumours. To clarify the roles of these gene expressions, six adult patients with pituitary adenomas treated in Tiantan Hospital from 2015 to 2020 and a pituitary adenoma cell line (Att20 sensitive to TMZ treatment) were analyzed by mRNA transcriptome sequencing. The differentially expressed genes were assayed by analyzing the sequencing results, and the expression level of these genes was further verified by immunohistochemistry. In addition, Ki67, VEGF, and p53 of the tumour tissues were also verified by immunohistochemistry.

**Results:**

In tumour tissues, mRNA sequencing showed that PTBP1 and EIF5A were significantly overexpressed in primary pituitary adenomas and SLC27A1 was significantly overexpressed in aggressive pituitary adenomas. Also in the pituitary adenoma cell line (AtT20), SLC27A1 expression levels were suppressed by TMZ treatment. Subsequent immunohistochemistry confirmed the sequencing results.

**Conclusion:**

High expression of SLC27A1 and low expression of EIF5A and PTBP1 may be potential indicators to predict the progression of aggressive pituitary adenomas, and patients with high SLC27A1 subtype may be sensitive to TMZ in clinical treatments.

## Introduction

Pituitary adenomas, one of the most common intracranial tumors, account for about 17% of intracranial tumors (1), non-functional pituitary adenomas account for nearly half of pituitary adenomas (2). Most pituitary adenomas show the growth characteristics of benign tumors, and most patients can be cured by surgery or drug treatments (2, 3). However, about 35% of pituitary adenomas show aggressive growth in imaging (4). These tumors grow rapidly and are resistant to conventional treatments such as surgery, medication and radiotherapy. This kind of tumor tends to relapse or regrowth in the early stage after operation (5, 6). We define this kind of tumor as aggressive pituitary adenoma. Non-functional pituitary adenoma recurred after operation is also a kind of aggressive pituitary adenoma (7). The literature reported that the postoperative recurrence rate of nonfunctional pituitary adenomas was 7.9%–46% (8, 9).

Temozolomide (TMZ) is a new alkylating agent, which interferes with gene transcription by methylation of DNA guanine and induces DNA damage. Temozolomide has broad-spectrum anti-tumor activity. Temozolomide is a first-line chemotherapy drug after the failure of the standard treatment of aggressive pituitary tumors. Patients who do not respond to TMZ treatment show resistance to TMZ. Therefore, there is no clear conclusion on the adaptation of temozolomide treatment. Blind use of TMZ treatment may waste resources, and may even affect the treatment of patients. Therefore, it is of great significance to explore the star gene of TMZ in patients with sensitive and aggressive pituitary adenomas.

Aggressive pituitary adenomas proliferate actively, oppress the optic nerve, and are not separated from the surrounding tissues, and are easy to invade the dura mater, cavernous sinus, bone, and other tissues, which brings difficulties to surgical treatment and affects the prognosis of patients (10). Therefore, it is of great significance to explore the factors affecting aggressive pituitary adenomas. Previous studies have shown that the Ki67 index, tumor invasion, and the extent of tumor resection are the factors affecting tumor progression (11, 12). According to the definition of WHO, the Ki67 index >3% in pituitary adenomas indicates active tumor proliferation (13, 14). Interestingly, some patients did not have a high Ki67 index but also showed tumor recurrence. On the contrary, some patients had a Ki67 index of more than 3%, but no tumor recurrence was found during long-term follow-up (11, 15, 16). Some studies have shown that p53 and VEGF are related to tumor invasiveness, but the relationship between p53 and tumor recurrence is still unclear. Therefore, these indicators are not accurate. It is particularly important to find the key genes to predict tumor progression from molecular biology.

## Methods and Materials

### Patients and Tumor Specimens

All the patient information is preliminarily determined through the database of Beijing Tiantan Hospital. The patients with primary pituitary adenomas were followed up for more than 5 years without recurrence (9). Patients with postoperative recurrence were defined as the aggressive pituitary adenomas group. All patients finished the examination of cranial contrast MRI and hormone levels before the operation. The clinical data of the research included clinical symptoms, radiographic examination, surgical results, immunohistochemical results, and follow-up results collected by reviewing clinical case information and telephone follow-up. The tumor specimens of each patient were preserved in two parts, and one of them was fixed with formalin immediately after the operation and embedded in paraffin blocks, the other part is sub-packed into a frozen tube and immediately stored in nitrogen liquid for mRNA analysis. Six tumor samples stored in liquid nitrogen were randomly selected for further transcriptional sequencing, including 3 patients with primary pituitary adenomas and 3 patients with aggressive pituitary adenomas. The wax blocks of 10 patients with primary pituitary adenomas and 10 patients with aggressive pituitary adenomas were randomly selected and stained for immunohistochemistry to verify gene expression.

This research was approved by the Ethics Committee of Beijing Tiantan Hospital. The procedures involving experiments on human subjects met the ethical standards of the Helsinki Declaration in 1975. All patients and their families signed up to participate in this study.

### Cell Culture and IC50 of TMZ

The mouse pituitary adenoma cell line GT1-1 (ATCC, USA) was cultured in Dulbecco’s modified Eagle medium (DMEM) containing 10% fetal bovine serum (FBS, Gibco, USA) and the mouse pituitary tumor cell line ATt20 (ATCC, USA) were cultured in 12K Medium (F-12K) supplemented with 15% horse serum and 2.5% FBS. Both GT1-1 and ATt20 cells were cultured at 37°C in a 5% CO_2_ humidified incubator.

Two types of cells were treated with different doses of TMZ (ranged from100 to 500 nM). The IC50 results for ATT20 cells are shown in Supplementary Material 1.

### RNA Sequencing and Bioinformatics Analysis

Primary and aggressive pituitary adenomas were chosen for RNA-seq according to intraoperative pathological diagnosis, Imaging diagnosis, and clinical diagnosis, each with 3 replicates. The clinical information of the tumor is shown in (Table 1). AtT20 cells treated with TMZ and mock-treated were tested for RNA-seq. (each with 3 replicates) RNA was extracted using TRIzol (Invitrogen, 15596-026, Grand Island, NY) according to the manufacturer’s protocol. Total RNA was reverse transcribed using an RT-PCR kit (Tiangen, KR103-03, Beijing, China) The sequencing readings are generated using the BGISEQ-500 platform as recommended by the manufacturer. Use tophat v2.0.12 to compare the paired-end clean reading with the reference human genome (UCSC version of hg19). The readings of each gene were calculated by HTSeq v0.6.1 and the gene expression level was calculated by RSEM v1.2.31. We use an MA map, volcano map, scatter map, and heatmap to express the distribution of different genes. The corrected *P-*value of Holm is 0.005 and the multiplier log2 (doubling) is 1 as the threshold for significant differential expression. Then the functional enrichment analysis of the gene was carried out.

**Table 1:** Clinical characteristics of patients whose tumor was detected by Transcriptome sequencing.

Patient ID	Gender	Age	Tumor location	Tumor size (cm)	Ki67 index (%)
P1	Male	27	Intrasellar	2.2	1
P2	Male	39	Intrasellar	1.7	1
P3	Female	56	Intrasellar + Suprasellar	1.2	3
R1	Male	53	Intrasellar + Suprasellar	3.4	3
R2	Female	45	Intrasellar + Cavernous sinus	3.6	3
R3	Male	39	Intrasellar	2.9	5
*P*-value	1.0	0.621	0.414	0.083	0.101

*P1–P3, Primary pituitary adenomas; R1–R3, Recurrent pituitary adenomas.*

### Differential Expression Gene Identification

The raw count expression matrix was normalized to RPM first. Identification of differential expression genes (DEGs) between TMZ with and without TMZ as well as DEGs between recurrent and primary pituitary adenoma patients was then completed using the limma R package (version 3.40.6) (https://bioconductor.org/packages/limma/). DEGs of the above experiment groups were extracted using eBayes method of limma with *P *< 0.05 as a cutoff value for a Benjamini-Hochberg adjusted *P*-value.

### Immunohistochemistry and Correlation Analyze

SLC27A1 (Solarbio, China, dilution:1:200), PTBP1 (Solarbio,China, dilution:1:100), EIF5A (Solarbio, China, dilution:1:50), Ki67 (Solarbio, China, dilution:1:100), VEGF (Solarbio, China, dilution:1:100), and P53 (Solarbio, China, dilution:1:200) were evaluated by immunohistochemistry. The paraffin-embedded specimens were cut into 4 μm thick sections. According to the manufacturer’s instructions, an antibody immunohistochemical analysis was performed on all sections using Leica Bond-III's automatic, random, and continuous slide staining system (Leica Biosystems, Germany). Images were obtained using a whole-slide scanner (3DHISTECH Ltd, Budapest, Hungary) and the immunohistochemical expression was scored by two observers using the CaseViewer software. The criterion of immunohistochemistry was that there were obvious brown granules in the cytoplasm or nucleus. The H score (range 0–300) was obtained by multiplying the staining intensity (0–3) by the percentage of positive cells (0%–100%), and the expression of tumor cells was scored semi-quantitatively.

### Statistical Analysis

The clinical and imaging data of all patients diagnosed with pituitary adenoma were mainly collected from the case and imaging system, as well as telephone follow-up. The follow-up period ranged from 4 to 168 months (mean 67 months). The descriptive statistics of the immunohistochemical results were expressed by the median and quartile range (IQR). The tumor size and Ki67 index between groups were compared by independent sample t-test. SPSS statistical software was used for the analysis. All statistical tests were two-tailed exact tests with a *P *< 0.05 considered significant.

## Results

### Differential Expression Gene Identification

**Figure 1:**
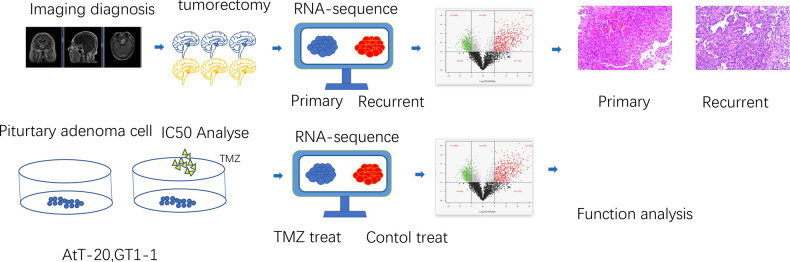
The process design of the experiment. At the tissue level, aggressive pituitary adenomas and primary pituitary adenomas were selected by clinical information and imaging data, and then pituitary adenomas were taken for transcriptional sequencing to screen the differentially expressed genes, and then the expression of differential genes was verified by immunohistochemistry. At the cellular level, pituitary adenoma cells were treated with TMZ to screen drug-sensitive cell lines, and then transcriptional sequencing was performed to screen differentially expressed genes for analysis.

**Figure 2:**
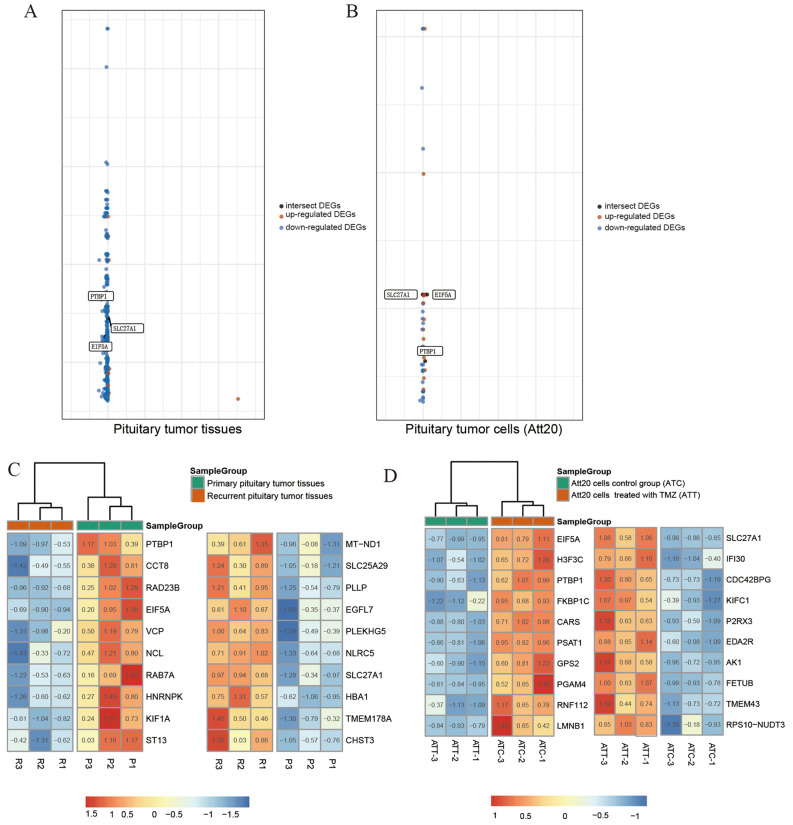
The graph shows the distribution of datasets with statistically significant mRNA overexpression (Brown) or down-regulated expression (blue) of the target gene. Genes intersecting tumor tissue (**A**) with cell sequencing (**B**) are labeled in black. The threshold was designed with the following parameters: *P*-value <0.001, fold change >1.5 (**A**,**B**). Sequencing heat map results of pituitary tumor tissues and cells are presented (**C**,**D**). Among the differentially expressed genes, the top 10 genes in the high expression ranking were each selected for heat map plotting with the top 10 genes in the low expression ranking. SLC27A1, eIF5A, and PTBP1 were expressed in both tumor tissues and cells.

The aggressive and primary pituitary adenomas and the ATt20 cells treated with TMZ were sequenced and analyzed with the normal control group (Figure 1). The differentially expressed genes were found by transcriptome sequencing analysis in aggressive and primary pituitary adenomas, and we selected the first 10 genes with the most significant differences between high expression and low expression (Figures 2A,C). After analysis, it is found that PTBP1, CCTB, RAD23B, EIF5A, VCP, NCL, RAB7A, HNRNPK, KIF1A, and ST13 is in the original. High expression in pituitary adenomas. And MT-ND1, SLC25A29, PLLP, EGFL7, PLEKHG5, NLRC5, SLC27A1, HBA1, TMEM178A, and CHST3 was highly expressed in aggressive pituitary adenomas. Pituitary adenoma cells Att20 were treated by TMZ and normal control cells were sequenced and analyzed by EIF5A, H3F3C, PTBP1, FKBP1C, CARS, PSAT1, GPS2, PGAM4, RNF112, LMNB1 was highly expressed in sensitive cell lines treated with TMZ, while SLC27A1, IFI30, CDC42BPG, KIFC1, P2RX3, EDA2R, AK1, FETUB, TMEM43, RPS10-NUDT3 was highly expressed in control cells (Figures 2B,D). After the data of the two groups were overlapped, it was found that PTBP1 and EIF5A were highly expressed in the primary pituitary adenomas and normal control pituitary tumor cells, and SLC27A1 was highly expressed in aggressive pituitary adenomas and TMZ-treated ATt20 cells.

### Immunohistochemical Analysis

**Figure 3:**
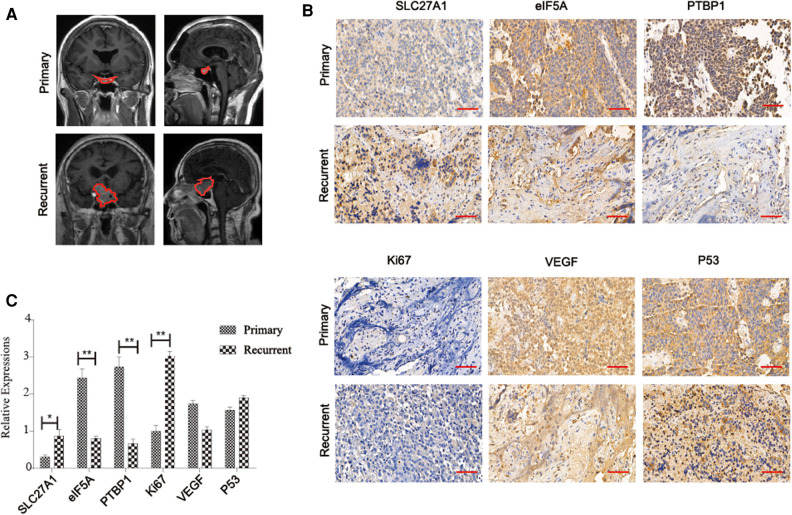
(**A**) Imaging information of primary and aggressive pituitary adenomas, the outline of the tumor is depicted with a red dotted line. (**B**) Immunohistochemical assay to verify the expression of SLC27A1, eIF5A, PTBP1, Ki67, VEGF, and p53 in recurrent and primary pituitary adenomas (**C**) Statistical analysis of immunohistochemical results

Immunohistochemical examination was performed in 10 patients with aggressive pituitary adenomas and 10 patients with primary pituitary adenomas. The results of representative immunohistochemical staining are shown in Figure 3. SLC27A1 was highly expressed in aggressive pituitary adenomas. Compared with aggressive pituitary adenomas, PTBP1 and eIF5A were highly expressed in primary pituitary adenomas. Ki67 was highly expressed in aggressive pituitary adenomas compared with primary pituitary adenomas. There was no significant difference between the expression of p53 and VEGF in aggressive pituitary adenomas and primary pituitary adenomas.

## Discussion

Recurrent nonfunctional pituitary adenomas account for more than half of aggressive pituitary adenomas. 57% of recurrent pituitary adenomas show invasive biological characteristics, which are more likely to invade the dura mater and bone, invade the cavernous sinus and enclose the internal carotid artery, which brings difficulties to surgical treatment and affects the prognosis of patients (17). Therefore, the study to predict the related factors of pituitary tumor recurrence is of great significance to improve the prognosis of patients. It is well known that clinicians should weigh a variety of factors to make individual treatment decisions. The purpose of this study was to explore the factors affecting the recurrence of pituitary adenomas from a genetic point of view.

Many clinical factors are affecting the recurrence of non-functional pituitary adenomas. In Brochier's research, it was found that tumor invasiveness was one of the prognostic factors affecting tumor recurrence (18). In our study, after a systematic analysis of patients, it was also found that tumors invading suprasellar, cavernous sinus and internal carotid artery were more likely to recur, and the difference was statistically significant. Similarly, the size of the tumor was also included in our study, and it was also found that tumors with a maximum diameter larger than 3cm were more likely to relapse. However, in the study of Lelotte et al, no correlation was found between the size of nonfunctional pituitary adenomas and tumor recurrence (10). Based on the results of our data analysis, we consider that invasive pituitary adenomas grow rapidly, invade surrounding tissues, and their tumors are larger in diameter because of their invasive growth characteristics. Of course, this requires more samples to verify its relevance.

The accuracy of predicting the recurrence of pituitary adenomas by the clinical characteristics of patients is poor. To improve the diagnosis rate, we further analyze the abnormally expressed genes in patients with aggressive pituitary adenomas from the perspective of genetics. It has been found that the expression of Ki67, P53, and VEGF is significantly increased in malignant tumors such as gastric cancer, bladder cancer renal cell carcinoma, and glioma (19–22). We speculate they are also abnormally expressed in aggressive pituitary adenomas. The features described as atypical adenomas in World Health Organization (WHO)’s classification in 2004 included Ki67 > 3%, increased mitosis, and extensive or strong expression of p53 (23). But these standards are quite controversial. Some resear ches have shown that the Ki67 index has a significant independent value in predicting recurrence (18), which is consistent with our results. But other studies have shown that this criterion is usually proved to be of predictive value only in the case of invasive tumors or residual tumors after surgery (10). No significant difference was found in the expression of p53 and VEGF in our study.

Therefore, the accuracy of the current molecules for predicting the recurrence of pituitary adenomas is poor, so it is more important to further look for abnormally expressed genes in aggressive pituitary adenomas.

Temozolomide is a new alkylating agent, which interferes with gene transcription by methylation of DNA guanine and induces DNA damage (24). Temozolomide has broad-spectrum anti-tumor activity and good permeability to the blood-brain barrier (25). At present, the clinical and basic research of TMZ in the treatment of aggressive pituitary adenomas is a hot spot, and scholars at home and abroad have carried out a series of studies on it. Losa et al reported a study of patients with aggressive pituitary adenomas treated with TMZ. During the treatment, 25 patients (80.6%) had pituitary adenomas under control and 6 patients (19.4%) had tumor progression. There is no definite conclusion on the indication of TMZ in the treatment of pituitary adenomas. Therefore, in order to find the sensitive genes which are effective in the treatment of TMZ, we carried out drug sensitivity test and sequencing analysis through the Att20 cell line.

Through a comprehensive analysis of the results of histological and cytological experiments, we found that the expression of SLC27A1 was significantly high in aggressive pituitary adenomas and TMZ therapy sensitive cell lines, while the expression of PTBP1 and EIF5A was significantly decreased. Studies have shown that SLC27A1 is involved in the regulation of lipid metabolism through the brain-liver axis (26), but the research in the field of oncology is still blank, so it is likely to be a new star molecule involved in the regulation of tumorigenesis and development. PTBP1 gene is involved in the regulation of glioma, multiple myeloma, and hepatocellular carcinoma (27–29), but its role in pituitary adenoma is still unknown. EIF5A was found to be abnormally expressed in thyroid carcinoma and breast cancer (30, 31). In pituitary adenomas, the subsequent immunohistochemical results confirmed the differential expression of SLC27A1, PTBP1, and EIF5A genes in aggressive pituitary adenomas and primary pituitary adenomas, and the difference was statistically significant. Therefore, SLC27A1, PTBP1, and EIF5A may be important molecular markers for predicting the recurrence of pituitary adenomas. Similarly, the results of cytological sequencing showed that the high expression of SLC27A1 suggested that TMZ treatment was effective, while the high expression of PTBP1 and EIF5A indicated that the therapeutic effect of TMZ was not good.

In summary, our study demonstrates that the high expression of SLC27A1 and the low expressions of PTBP1 and EIF5A predict the progression of an aggressive pituitary adenoma. Data from murine pituitary adenoma cell lines AtT20 together with TMZ-sensitivity tests suggest that aggressive pituitary adenomas with this molecular pattern may be responsive to TMZ treatment.

## Data Availability

The datasets presented in this study can be found in online repositories. The names of the repository/repositories and accession number(s) can be found in the article/Supplementary Material.
