# Supplementary figures and images for: Identification and Verification of SLC27A1, PTBP1 and EIF5A With Significantly Altered Expression in Aggressive Pituitary Adenomas
Source: Front Surg. 2022 Jun 21;9:923143. doi: 10.3389/fsurg.2022.923143 (PMC9275011; doi:10.3389/fsurg.2022.923143)

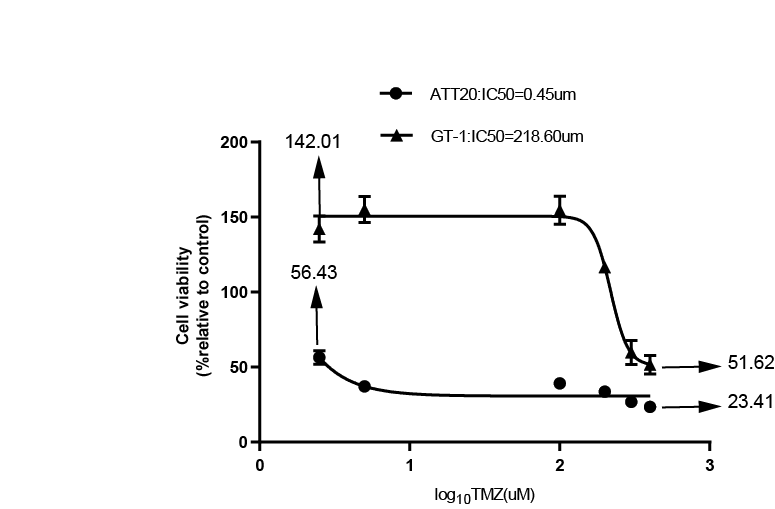

Supplement: Supplementary file 1 [file Image_1_v1.tif]
